# How to compare movement? A review of physical movement similarity measures in geographic information science and beyond

**DOI:** 10.1080/15230406.2014.890071

**Published:** 2014-03-07

**Authors:** Peter Ranacher, Katerina Tzavella

**Affiliations:** ^a^ Department of Geoinformatics – Z_GIS, University of Salzburg, Schillerstraße 30, 5020Salzburg, Austria; ^b^ Fraunhofer FKIE, Fraunhoferstr. 20, 53343Wachtberg, Germany

**Keywords:** movement comparison, similarity measures, moving objects

## Abstract

In geographic information science, a plethora of different approaches and methods is used to assess the similarity of movement. Some of these approaches term two moving objects similar if they share akin paths. Others require objects to move at similar speed and yet others consider movement similar if it occurs at the same time. We believe that a structured and comprehensive classification of movement comparison measures is missing. We argue that such a classification not only depicts the status quo of qualitative and quantitative movement analysis, but also allows for identifying those aspects of movement for which similarity measures are scarce or entirely missing.

In this review paper we, first, decompose movement into its spatial, temporal, and spatiotemporal movement parameters. A movement parameter is a physical quantity of movement, such as speed, spatial path, or temporal duration. For each of these parameters we then review qualitative and quantitative methods of how to compare movement. Thus, we provide a systematic and comprehensive classification of different movement similarity measures used in geographic information science. This classification is a valuable first step toward a GIS toolbox comprising all relevant movement comparison methods.

## Introduction

How similar do two or more objects move with respect to one another? This is an important question in many fields of science in general and in geographic information science in particular (Laube et al. 2007; Vlachos, Gunopulos, and Das 2004). Accordingly, various studies on movement comparison can be found in literature: Dodge, Laube, and Weibel (2012) cluster hurricanes that have reached the shore of the United States between 1907 and 2007 based on the similarity of the hurricanes’ movement across the Atlantic Ocean. Waddington (1979) analyzes three breeds of bees in order to detect similar foraging behavior. Kang et al. (2010) compare the movement of mobile phone users in China. They describe to what degree the mobility patterns of certain age and gender groups differ from one another. Gavric et al. (2011) analyze geo-referenced photos from the online photo sharing platform Flickr that were uploaded by visitors of the city of Berlin. The researchers connect the coordinates of the photos of a single user to spatiotemporal trajectories. Then they cluster similar trajectories to derive those routes in Berlin that are most frequented by tourists who post on Flickr. Interestingly enough, even though all the aforementioned authors aim at quantifying the similarity of their moving objects under study, they do not share a universal concept of *how* to assess this similarity. Quite the contrary is true. Different authors compare movement with utterly different methods on utterly different physical levels. For Dodge, Laube, and Weibel (2012) two hurricanes move similarly if their paths have similar phases of speed and change of direction of movement. Waddington (1979) considers bees to move similarly if they cover an equal flight distance and change their direction of flight from one flower to another in a similar fashion. For Kang et al. (2010) similar movement of mobile phone subscribers refers to similar average travel distances. Gavric et al. (2011) consider that two tourists move similarly if their paths coincide and connect touristic sights in the same spatial progression. Here, we mention only four different methods on how to assess the similarity of movement, whereas – theoretically and practically – there are a lot more. We want to illustrate this with an example.

**Figure 1.**
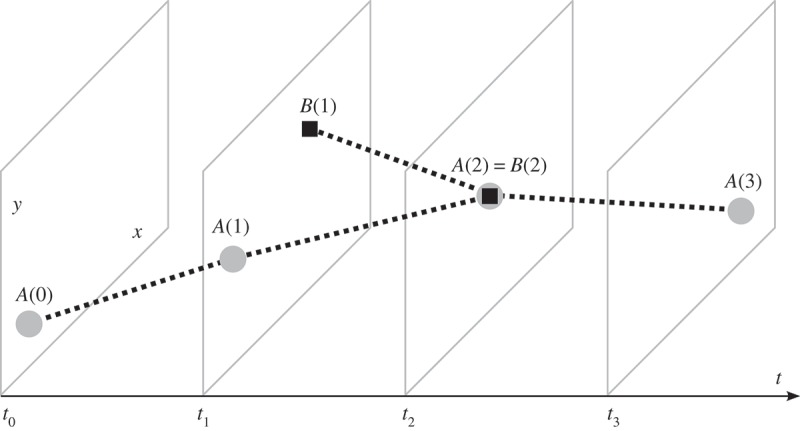
Two moving objects in two-dimensional space (*x*- and *y*-axis) and time (*t*-axis).

In Figure 1, the circle and the square represent two moving objects. At time 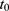 the circle is at location 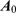. It moves to location 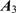 where it arrives at time 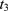. On its way, it passes the positions 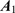 and 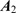. The square starts its movement at location 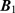 at time 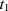. It moves to location 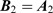 where it arrives at time 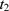. Now, how similarly do the two objects move? In order to answer this question we have to first specify the term similarity.

Lin (1998) defines an intuition for similarity as follows: the more commonality two objects share the more similar they are. Consequently, the more differences they have the less similar they are. The maximum similarity occurs when the two objects are identical.

Now we may take a closer look at movement and its physical quantities, as these are our different ‘levels’ to assess similarity. Without doubt movement bears a temporal dimension; hence one might be interested in comparing movement from a temporal point of view. The circle starts moving before the square and stops after it. Consequently, one conclusion is that the two objects partly move at the same time, in a way that the square is moving during the time when the circle is moving. Accordingly, one might want to know, whether the movement of the two objects is similar from a spatial point of view, as well. In Figure 1, the spatial paths of the circle and the square intersect at 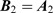. Moreover, the two objects attend this position at the same time. Therefore, not only the paths but also the spatiotemporal *trajectories* of the two objects intersect. Hence, we compare movement from a spatiotemporal perspective.

From the example above it may be concluded that movement has a temporal, a spatial and a spatiotemporal dimension. Accordingly, this paper aims at decomposing movement into its physical quantities in time, space, and space-time. Each of these quantities represents one level for which we review measures on how to compare the similarity of movement. In addition to these physical properties of movement, there is also an ‘intrinsic dimension’ of movement: an object moves for a specific purpose, to meet a specific need or fulfill a specific task. Intrinsic movement similarity is briefly discussed where it complements physical similarity, but is generally not part of this paper.

It is quite impossible to cover the entirety of approaches that has been developed in order to assess the similarity of moving objects in a single review. The comparison of movement is important in different fields of science – ranging from biology, to sociology and geography – to name but a few. These fields and their objects under study require very specific similarity measures that are often heavily tailored to the problem under consideration. This results in a plethora of different similarity measures that exist in literature. Nevertheless, we understand our paper as a first step toward a collection of movement similarity measures – that is not complete, but as complete as possible.

The remainder of the paper is organized as follows. Section ‘Related work’ provides an overview on the current state of movement analysis. Section ‘The physical quantities of movement’ decomposes movement into its physical quantities and shows how these quantities are related to each other. Section ‘Comparing movement at different levels’ reviews the most important measures for assessing similarities between movements at different physical levels. Section ‘Summary and conclusion’ summarizes and concludes the results. Section ‘Discussion and future work’ presents the discussion and an outlook on future work.

## Related work

Today’s presence of ubiquitous positioning devices allows for collecting detailed traces of movement in space and time. These traces represent a novel data source that requires novel methods for analysis, one of them being measures to assess movement similarity. In this section we discuss literature on movement similarity as well as its relations to other aspects of movement analysis. First, we account for the fact that usually not movement itself but a representation of movement (i.e. a recording of movement) is compared. Then we discuss the quality of these recordings and the influence of the spatial accuracy, sampling rate and uncertainty. Last, we present work that aims at collecting and summarizing methods of movement similarity analysis.

### Representing movement

A moving object is any identifiable entity that moves and exists independent of other objects (Macedo et al. 2008). Güting and Schneider (2005) distinguish between two fundamentally different classes of moving objects: objects that maintain a constant shape while moving (e.g. a human being, a vehicle, an animal) and objects that change their shape (e.g. a forest fire). Conceptually, the former are mostly represented as simple point elements, whereas the latter require polygons to model their time-dependent change in extent. As for this paper we exclusively concentrate on similarity measures for point objects.

Movement describes the change of the object’s position in a spatial reference system with respect to time. In real world, change is per se continuous (Sinha and Mark 2005). When a moving object is recorded (e.g. by a Global Positioning System (GPS) logger), only discrete snapshots of the object’s whereabouts are captured and preserved. Andrienko et al. (2008) distinguish between five strategies of how to record snapshots of movement: time-based (a snapshot is recorded after a regular time interval), change-based (a snapshot is recorded when the object changes its position), location-based (a snapshot is recorded when an entity is near a certain spatial location), event-based (a snapshot is recorded when a certain event occurs), and various combinations of these. Depending on which method is used, the same real movement may be represented in entirely different ways.

The resulting representation of movement is called a discrete trajectory. Even though discrete trajectories comprise a non-continuous series of spatiotemporal positions, interpolation can be used to approximate the original, continuous movement. In this case trajectories can be seen as continuous functions from time to space (Andrienko et al. 2008). The fastest and easiest interpolation method is piece-wise linear interpolation (Macedo et al. 2008): a simple straight line connects each two consecutive recorded positions. Along this line the moving object is assumed to move at constant speed. Changes of speed and direction occur abruptly at each position measurement. This is to some extent contrary to real movement where speed and direction change smoothly and gradually. Thus, linear interpolation is not the only way of restoring continuous movement. Other interpolation methods include cubic or high-order polynomial interpolation (Lin, Chang, and Luh 1983). These aim to overcome the shortcomings of linear interpolation.

### Entire lifelines and subsequences of movement

In general, most moving objects are dynamic with respect to their surroundings over the whole period of their lifespan. Consequently, Mark and Egenhofer (1998) term trajectories as geospatial lifelines that ‘describe the individual’s location in geographic[al] space’. Different parts along this lifeline are associated with different semantics (Parent et al. 2013). As for living beings, a change in location corresponds to meeting a need: living beings look for food, for a safe place, or a member of the same species to reproduce. Each of these activities lends movement a meaning or purpose. When comparing the movement of two objects, researchers are more often than not interested in assessing the similarities of meaningful subsequences of movement rather than comparing entire geospatial lifelines (Buchin et al. 2009). We want to illustrate this with an example.

When tracking the movement of an albatross (cf. Edwards et al. 2007), the avian lifeline is recorded as soon as the positioning device – in this case a GPS receiver – is attached to the seabird and switched on. Correspondingly, the trajectory ends when the positioning device is switched off and removed from the bird. The domain-expert – i.e. the ornithologist – defines those breakpoints that divide the entire lifeline into legs of specific purpose. For an albatross, a purpose of movement is foraging or migration; therefore, respective breakpoints are stopovers on the ground or departure and return to a nesting habitat. Consequently, the researchers analyze the sub-trajectories that represent foraging or migratory behavior, rather than the entire lifeline of the bird (Spaccapietra et al. 2008). The definition of breakpoints and meaningful sub-trajectories depends on the aim of the research, contextual information as well as expert knowledge. Trajectory segmentation is concerned with finding objective criteria and methods to split entire lifelines into meaningful segments (cf. Buchin et al. 2011; Buchin, Kruckenberg, and Kölzsch 2013).

### Spatial accuracy, temporal resolution, and spatial uncertainty

Trajectories allow for recording, representing, and storing the behavior of moving objects in space and time. Moreover, they build the conceptual fundament for movement comparison: we do not assess how similarly two objects move according to their actual behavior in space and time, but according to their measured representation. This implies that the methods used for collecting trajectories have a fundamental impact on similarity assessment. In particular this applies to the spatial accuracy of the measurement device and the temporal resolution of recording. In geographic information science, spatial accuracy defines how closely a measured position matches the real position of a geographic feature in space (Chang 2008). The mean spatial accuracy of a standard GPS receiver, for example, equals 3 meters horizontally at a 95 % confidence interval.^1^

Temporal resolution, on the other hand, refers to the update rate of measurements (Longley et al. 2005). For trajectories, it is the time span between recording each two consecutive positions. Temporal resolution affects the spatial path of the trajectory and its uncertainty. Only at measured positions the whereabouts of the moving object are known, the interpolations between these are basically a *guess* about the actual movement. In general, the lower the temporal resolution, the less certain the whereabouts of the moving objects are known (Pauly and Schneider 2004). In order to quantify spatial interpolation error, Pfoser and Jensen (1999) introduce the concept of uncertainty trajectories. When the maximum possible speed of a moving object is known in advance, several paths may allow the object to leave a known position and arrive at the consecutive one in the allotted time. The union of all these possible paths projected to two-dimensional space results in the so-called uncertainty ellipse. Uncertainty ellipses are defined by two parameters: the maximum speed of the moving object and the temporal resolution of position fixing. Hence, they can be used to guarantee that the spatial uncertainty of the trajectory stays beyond a certain boundary (Ranacher and Rousell 2013). Uncertainty ellipses are valid for unconstrained movement. For an object that is restricted to a network, such as a car to a road network, other methods are applied that re-engineer the most probable path that the object has followed (Zheng et al. 2011).

The fact that sensor measurements are affected by low sampling and error is addressed in the state estimation and target tracking literature (Bar-Shalom, Li, and Kirubarajan 2004; Koch 2010). The approach by Tzavella and Ulmke (2013) utilizes and combines output from particle filtering tracking (sequential Monte Carlo) and GIS techniques. The goal is to infer the actual path of a moving object from sensor measurements which suffer from limited resolution, measurement noise, false alarms, and missed detections due to small target velocity or terrain shadowing.

### Movement comparison and movement patterns

Movement pattern analysis is a research field closely related to movement comparison and similarity assessment. Dodge, Weibel, and Lautenschütz (2008) define a movement pattern as ‘a regularity in space or time or any noteworthy relation between movement data’. Movement patterns can be divided into two main classes: they either describe the movement behavior of a single moving object or the relation of two or more moving objects to each other (Jeung, Yiu, and Jensen 2011). Clearly, both types of patterns rely on movement comparison. For finding individual patterns, an object’s movement is compared to itself over time. For group patterns two or more objects are compared against each other. We want to illustrate this with two examples. The individual movement pattern *constancy* requires that a moving object has a movement parameter that is invariant over time (Laube, Imfeld, and Weibel 2005). The individual pattern ‘*constancy of speed*’ can be rephrased as a simple comparison: ‘Which objects exhibit a similar speed during their entire movement?’ The group pattern *moving cluster* requires objects to move close to one another for a certain time span (Gudmundsson and van Kreveld 2006; Kalnis, Mamoulis, and Bakiras 2005). In order to detect whether two objects qualify as a *moving cluster*, their paths have to overlap and occur at the same time. A structured overview on movement patterns can be found in Dodge, Weibel, and Lautenschütz (2008).

### Movement comparison

An extensive literature review on movement similarity measures is presented by Dodge (2011) in the form of an introductory section to a PhD thesis. However, this review mainly focuses on quantitative measures. Purely qualitative measures are not covered. Long and Nelson (2012) review qualitative and quantitative methods for analyzing movement data. They briefly discuss the topic of movement similarity, their main focus, however, lies on a general review of movement analysis. Other – more or less extensive – reviews of movement similarity measures are often found in the related work section of articles that introduce novel similarity measures. Frentzos et al. (2008) provide a short overview on similarity research for trajectories and mention the need for further similarity measures. Dodge, Laube, and Weibel (2012) divide methods for assessing the similarity of moving objects into two classes: spatial similarity and spatiotemporal similarity. Spatial similarity methods fall back on the spatial path and its shape as the only comparable measures to check whether two trajectories are similar; accordingly, spatiotemporal similarity methods compare movement with respect to spatial as well as temporal aspects. In spite of all the literature mentioned above, to the best of our knowledge an exhaustive literature review is missing that

- focuses on the classification of movement similarity measures;
- distinguishes between qualitative – or topological – and quantitative approaches;
- and explains for which data sets and tasks the measures are used.

## The physical quantities of movement

**Figure 2.**
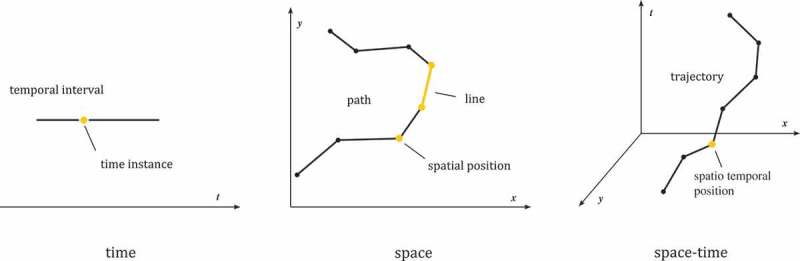
Primary movement parameters in time, space, and space–time.

Dodge, Weibel, and Lautenschütz (2008) propose a set of characteristic features of movement, which they refer to as movement parameters. A movement parameter is an inherent physical quantity of movement, such as the duration of the movement or its speed. Similar to Dodge, Weibel, and Lautenschütz (2008), we decompose movement into its physical quantities. These represent the different levels at which movement is compared. Movement parameters are either primary ones and refer to a distinct position in an absolute reference system, or derived and indicate the relative change between two primary parameters. Consequently, primary movement parameters are measured, whereas derived movement parameters are calculated from one or more measurements. Figure 2 shows all primary movement parameters. The distinction between primary and derived movement parameters is important for finding applicable measures of how to compare movement and how to interpret their results. The following section recaps the most important primary and derived movement parameters.

### Temporal movement parameters

Temporal movement parameters describe *when*, *for how long*, *how often*, and *how regular* an object is moving. The principal measurement in the temporal dimension is a *time instance* (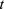). *Time instance* reflects an infinitesimally small point in time at which a moving object exists. An ordered list of *time instances* is referred to as a *temporal interval* 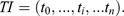 A *temporal interval* increases strictly monotonically and has infinitely many elements (Venema 2001). It contains all *time instances* at which the object is moving. *Time instance* and *temporal interval* are primary movement parameters (see also Figure 2).

A *temporal duration* 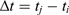 is the time difference between two *time instances*, where the latter is supposed to occur earlier in time than the former. A *temporal duration* describes the amount of time an object is moving; it is a derived movement parameter.

### Spatial movement parameters

Spatial movement parameters describe *where, how far*, and in which *direction* an object is moving. The principal spatial observable is a *spatial position* that a moving object attains. In two dimensions, a spatial position is defined as 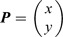. A *spatial path* 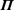 describes the spatial progression of movement. It is an ordered list of actually measured spatial positions: 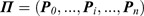; each two consecutive positions are connected by a (well-defined) interpolation function. For the case of linear interpolation, the *line* between each two spatial positions is defined as 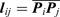. *Spatial position*, *line*, and *path* are primary movement parameters (see also Figure 2).

The *position difference* 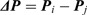 refers to the relative difference vector between two *spatial positions* (Hofmann-Wellenhof, Legat, and Wieser 2003). The Euclidean distance represents the length of this vector: 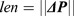. The unit vector of 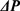 is the *direction* (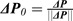) between the two *spatial positions*.

In order to describe the distance between two positions along a spatial path two different distance concepts are applied: the *range* between two positions 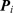 and 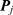 refers the distance along the straight line difference vector; *travelled distance* refers to the distance along the moving object’s path. If we consider the positions to be connected by piece-wise linear interpolation, travelled distance equals the sum of all spatial difference vectors between 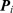 and 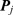. From this we can conclude that travelled distance highly depends on the temporal sampling rate at which movement is recorded: the higher the sampling rate, the longer the resulting path. This relates to the problem of the length of the coast of Britain raised by Mandelbrot (1967).

The sum of all consecutive *position difference* vectors results in the *shape* of the *spatial path*. Shape is independent of an absolute position in a reference system. It can be expressed by other derived parameters such as *sinuosity*, *curvature*, *tortuosity, curviness*, *or fractal dimension*. Each of these – in some way or the other – depicts the degree of ‘winding’ of a path. *Sinuosity*, for example, relates *travelled distance* to *range*. For a detailed definitions of *sinuosity, curvature, curviness*, and *tortuosity*, see Buchin et al. (2011). *Fractal dimension* measures to what degree a path ‘fills’ the space it is roaming in (Mandelbrot 1983): a straight line fills space least, whereas an entirely random motion fills it most.

### Spatiotemporal movement parameters

Each *spatial position* is recorded at a specific *time instance*. Hence, the spatial and temporal observables can be combined into a single expression, a *spatiotemporal position* 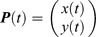. A *trajectory* 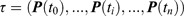 is an ordered sequence of *spatiotemporal positions*. *Spatiotemporal position* and *trajectory* are primary movement parameters (see also Figure 2).

*The velocity* vector 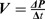 captures the relative motion of an object between two *spatiotemporal positions* (Hofmann-Wellenhof, Legat, and Wieser 2003). The length of the *velocity* vector is the *speed* 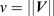 of the moving object. The unit vector of *velocity* indicates the *heading* of the object (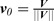). Geometrically, *heading* and *direction* are equal. Henceforth, we refer to both as *heading*. *Velocity, speed,* and *heading* are derived parameters.

The *acceleration* vector 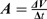 captures the change of *velocity* over time. The length of the *acceleration* vector is the *change of speed* over time: 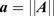, also referred to as *acceleration* (*scalar*). The unit vector of the *acceleration* vector indicates the *change of heading* (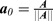). *Acceleration* (both vector and scalar) and *change of heading* are derived parameters.

## Comparing movement at different levels

This section reviews the most important concepts of how to compare the movement of two or more objects. Each physical quantity of movement discussed in section ‘The physical quantities of movement’ represents one level of comparison. In addition to these we introduce three criteria that define the type of similarity measure.

### Types of similarity measures

The following three criteria are used to distinguish between different types of similarity measures:

- Is the measure applicable for primary or derived movement parameters?
- Does the measure rely on a topological or quantitative comparison of movement?
- What is the measure intended and/or mainly used for?

The three criteria are discussed in this section together with the types of similarity measures they define.

#### Similarity measures for primary and derived movement parameters

In section ‘The physical quantities of movement’ we distinguish between primary and derived movement parameters. Consequently, we also divide similarity measures into those for primary movement parameters and those for derived movement parameters. For simplicity these are henceforth referred to as primary and derived similarity measures. Primary similarity measures compare the movement of two objects with respect to their positions in a temporal, spatial, or spatiotemporal reference system. An example for a temporal reference system is the Gregorian calendar, a spatial one is the World Geodetic System 1984 and a spatiotemporal the space–time cube (see also, Kraak 2003). In these reference systems, two objects might move similar to each other with respect to (i) time, (ii) space, or (iii) space–time. For (i) they share the same spatial path, for (ii) they move at the same time, for (iii) they share the same path at the same time. In other words, movement that is similar with respect to its primary parameters occurs at similar times or occupies similar space. Correspondingly, derived similarity measures compare movement with respect to those characteristics that are independent of a spatiotemporal reference frame. Two objects might move for the same duration or have a similar speed without sharing similar paths or moving at the same time.

#### Topological and quantitative similarity

The second criterion classifies a measure as topological or quantitative. According to Price (2013), topology is concerned with the study of qualitative properties of certain objects. It is a mathematical concept that allows for structuring data based on the principles of feature adjacency and feature connectivity. A topological relation is preserved if the object is rotated, scaled or translated (Rinzivillo, Turini, et al. 2008). Topological relations may also be termed qualitative relations. However, the key publications reviewed for this paper mostly use the more specific term topological relations. Hence, this term is also adopted in this paper. When a qualitative relation does not qualify as a topological one, this is mentioned specifically. For two moving objects, topological similarity measures describe how the movement parameters of these objects relate to each other without taking into account any quantitative consideration. Thus topological similarity measures help to answer questions such as: ‘Do the spatial paths of the objects intersect?’, ‘Do the objects move during the same time?’, ‘Do the objects move away or towards one another?’

Quantitative similarity allows for expressing relations of two moving objects in terms of numbers that can be calculated or measured. Thus, it allows for answering questions such as ‘How far are the objects away from each other in space?’, ‘How close are the trajectories of these objects in space and time?’ Quantitative or non-topological similarity is usually associated to a distance function. Distance functions are either metric or non-metric. A metric distance function 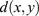 satisfies the following four axioms; it is

- non-negative 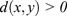;
- unique 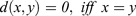;
- symmetric 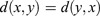;
- and satisfies the triangle inequality (Chaudhuri and Rosenfeld 1996).

Simple *Euclidean distance* is an example for a metric measure. A non-metric measure is the *longest common subsequence* (*LCSS*) described in section ‘Spatiotemporal trajectory’.

#### Purpose of the similarity measure

This criterion defines the purpose for which the similarity measure is intended or mainly used for. We distinguish between four types of purpose:

- description – the measure explains or formalizes a relation between the two moving objects;
- clustering – the measure is used to group similar moving objects;
- similarity search – the measure finds most similar moving object with respect to a reference object;
- behavior analysis – the measure describes the behavior of one object with respect to another;
- outlier detection: the measure identifies unusual behavior in a set of data.

Of course, these criteria are overlapping and should not be understood as exclusive. A measure that is used for clustering also allows performing a similarity search. Moreover, it is based on a formalized relation between two movement parameters.

In addition to these three criteria, we give examples of data sets to which the respective measure is or may be applied. Moreover, we add the computational complexity of the measure: low refers to linear or quasilinear complexity, medium to quadratic complexity, and high to polynomial or higher complexity. However, this classification is neither meaningful nor possible for all measures. First, some measures are only defined theoretically and are not implemented algorithmically. For these computational complexity is not explicitly mentioned. Second, for some measures there exist heuristics that may considerably improve the computational complexity, but retrieve non-optimal results. In addition to this, complexity may relate to the comparison of an entire data set (i.e. clustering), or to the comparison of two entities in the data set.

In the following section the different similarity measures are discussed.

### Temporal similarity measures

Temporal similarity measures are based on either a linear or a cyclic concept of time (Luisi 1999): linear time flows continuously from the past to the future. *Time instances* refer to an exact position along this time flow, similar to a number on a number ray. Consequently, two time instances are equal if they occur at the same position along this time flow. Any arbitrary time instance may serve as an origin for a temporal reference system based on linear time. For example, GPS uses the *time instance* 0h UTC, January 5 1980 as a time zero point (Lewandowski and Thomas 1991). If time is considered cyclic, it is assumed to ‘repeat’ after a certain temporal interval. This interval is most intuitively related to the Earth’s rotation around its own axis (day) or the sun (year); other intervals follow human concepts related to Earth rotation (week, month, decade). In cyclic time, two time instances are equal if they occur at the same temporal position during one cycle, i.e. if a well-defined interval has passed between them: whereas January 1 2012 is distinct from January 1 2013 in linear time, these dates are equal in a time concept based on the annual cycle.

#### Time instance

Time instances are positions in a temporal reference frame; hence they require primary similarity measures. A topological relation between two time instances 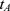 and 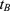 is trivial: they either *intersect*, or do *not intersect*. If time instances do not intersect, one occurs *before* or *after* the other. Hodgson et al. (2006) analyze the migration of different populations of salmon from the ocean back to their birth place in the rivers of Alaska. They conclude that specific salmon populations enter their riverine habitat *before* others. Kjellén (1992) studies the autumn migration of raptors from Sweden to the tropics. He finds that adult honey buzzards migrate *after* their juvenile conspecific. Both studies essentially use a topological comparison of *time instances* or events during migration that bear a specific meaning for the two species: the *time instances* when the salmons return to and the raptors leave their home habitat.

The *temporal distance* between 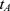 and 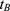 is a quantitative measure and refers to the amount of time between two events in time. In their work on salmon migration Kovach et al. (2013) conclude that climate change causes salmons to start migrating *1.7 days earlier* in comparison to former decades. This *temporal distance* is related to a cyclic notion of time: the time instances that mark the beginning of salmon migration are compared according to their occurrence in the course of a year.

#### Temporal interval

**Figure 3.**
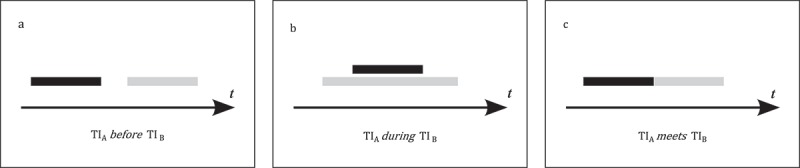
Three examples for Allen’s temporal logic (based on Allen 1983).

In contrast to zero-dimensional time instances, temporal intervals cover a certain temporal extent; they are essentially one-dimensional line objects in one-dimensional temporal space. Therefore they require slightly more complex topological relations. Allen proposes a qualitative *temporal logic* with a complete set of 13 distinct relations between two temporal intervals (Allen 1983). Figure 3 shows three temporal relations between 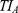 (black) and 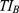 (gray): (a) 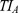 *before* 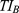, (b) 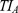 *during* 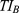, and (c) 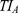 *meets* 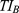. For a complete set of all 13 relations, see Allen (1983). Allen’s temporal logic is a primary similarity measure and may describe the topological similarity of movement in time. The computational complexity of the different relations is discussed in detail in Golumbic and Shamir (1993).

Fox, Glahder, and Walsh (2003) analyze the spring migration of geese from Ireland to Greenland. Their data suggest that some birds start their passage earlier in autumn and arrive to Greenland later than other birds. Translated into Allen’s temporal logic, the migratory movement of the ‘faster’ birds occurs *during* that of the ‘slower’ ones.

Each time interval comprises a start and an end time instance. The *temporal distance* between respective end and start time instances results in a quantitative measure of how the temporal intervals are different. In the above-mentioned research on migratory geese, one ‘fast’ goose leaves Ireland *three days after* another ‘slower’ one, but reaches the shores of Greenland about *ten days before*.

#### Temporal duration

Temporal duration refers to the time span of one meaningful leg of movement. Temporal duration is a derived measure. In a qualitative (topological) way, two durations can be compared with the well-known set of *relational operators* ‘*=*’ (equal duration), ‘*<*’ (shorter duration), and *‘>’* (longer duration). A quantitative measure is the *difference between two durations*. Ueta et al. (2000) track the movement of adult and juvenile sea eagles. They find that the migratory movement of adults lasts *shorter* than that of their younger conspecific.

### Spatial similarity measures

#### Spatial position

The topological comparison of two *spatial positions* is trivial: the two positions either *intersect* or do *not intersect* (Egenhofer and Herring 1991). Girardin et al. (2008) analyze the spatial occurrence of mobile phone calls to reason about the movement of tourists in the city of Rome. A tourist’s mobile phone call stands for one discrete spatial and temporal presence of the tourist. Wherever a sufficient number of tourists are sensed, the researchers identify a touristic hotspot. A hotspot is essentially a location in the city of Rome, where the call positions of many tracked tourists *intersect*. In avian migration, stopover locations represent one important spatial position along the birds’ migratory path. In a study on crane passage from Russia to China, Higuchi et al. (1996) find that the demilitarized zone between North and South Korea hosts a major stopover site for their birds under study. Here, the individual stopover locations of the birds *intersect*. (Note: whether a hotspot is interpreted as a point or an area largely depends on the aim of the analysis and on scale. For reasons of simplicity, here, a hotspot is viewed as a point.)

A quantitative relationship of spatial positions is the *spatial distance* between them. A *spatial distance* function describes how far two points are away from each other in space. Obviously, spatial distance strongly relies on the underlying reference system, its characteristics and dimensionality. Intuitively, the most common distance function is *Euclidean distance*, which describes the length of the straight line between two points in Euclidean space. Euclidean distance is, but a special case of the more general *Minkowski distance*. *Minkowski distance* is calculated as 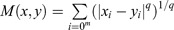. For 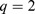 the Minkowski distance equals the Euclidean distance, for 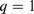 the grid-like *Manhattan distance* (Perlibakas 2004).

Distance measures for reference systems other than Euclidean, comprise *distances along curved surfaces* (such as the *spherical distance* on a globe and the *spheroidal distance* on an ellipsoid), or *network distances*. In a network, a cost function represents the effort it takes to pass a path between two nodes. The cost value might refer to the length of that path in terms of Euclidean distance, as well as the time or an abstract cost needed to traverse the path (Hofmann-Wellenhof, Legat, and Wieser 2003). In a road network, costs may – for example – represent a car’s expected fuel consumption (Minett et al. 2011). Depending on the cost function, network distance is a metric (*Euclidean distance*) or a not a metric (e.g. *fuel consumption*).

In two-dimensional Euclidean space a moving object has two degrees of freedom. Consequently, spatial distance is not the only measure of how to compare two spatial positions: we lack information on the *spatial direction* of this distance. In Euclidean space, direction is expressed as the unit vector of the distance vector between the two positions. The *relative direction* of the unit vector with respect to a reference vector (e.g. a coordinate axis) yields a quantitative angular measure (e.g. 90°) (Hofmann-Wellenhof, Legat, and Wieser 2003). Frank (1996) introduces a qualitative – but not topological – method for comparing directional information based on the *cardinal directions* in a compass. He suggests different approaches to partition space based on the observer’s position: cardinal directions of cones (North, West, South, East), of half planes (Northwest, Northeast, Southwest, Southeast) and directions with a neutral zone at the location of the observer (North, Northwest, West, Southwest, South, Southeast, East, Northeast, and neutral zone) (Frank 1996).

In an analysis on avian migration Chevallier et al. (2011) identify the stopover locations of black storks on their flight from Europe to Africa in autumn and vice versa in spring. The researchers find that the stopover locations of individual birds do not match for spring and autumn migration. For instance, the tracked bird named Aurelia has its longest spring stopover in Spain approximately 83 km (spherical distance) North of its autumn stopover.

#### Spatial path and line

The *9-intersection* model describes the different topological relationship between the interior (), the boundary (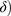, and the exterior (–) of two simple lines in two-dimensional space (Egenhofer and Herring 1991). According to definition, a simple line has a boundary that consists of exactly two points of zero extent. A path comprises exactly one starting and one end position. Hence, a path in two-dimensional space qualifies as a simple line. Egenhofer and Herring (1991) propose 33 distinct relations between two simple lines.

Depending on the measurement device and the sampling strategy a qualitative comparison of spatial paths might be unrealistic. Two paths recorded by GPS in a time-based manner will hardly ever exactly intersect. In order to allow for qualitative analysis the underlying space has to be discretized. Locations that are spatially close are aggregated into one area; topologically this area is then again treated as one single spatial position. On the one hand, a discretization of space may follow from the sampling strategy used for recording movement. Two examples for sampling strategies that discretize space are event-based recordings in a mobile phone network (cf. Calabrese et al. 2011; Calabrese et al. 2010; Girardin et al. 2008; González, Hidalgo, and Barabási 2008) and location-based recording with Bluetooth scanners (cf. Versichele et al. 2012). In both cases the position of the static sensor (base transceiver station or Bluetooth scanner) is used to indicate the position of an object that is close to the sensor. Thus the network itself divides space into discrete areas comprising the sensor and its vicinity (in a mobile phone network referred to as a cell). On the other hand researchers might discretize space into areas according to knowledge gained from the movement (Andrienko et al. 2011; Andrienko and Andrienko 2010) or knowledge they have about space (e.g. due to territorial units).

Independent of similarity in real space, movement may occupy an abstract feature space (cf. Andrienko et al. 2013). Abstract space is relevant in the field of human activity recognition, i.e. research aiming at inferring human activities from movement traces (Furletti et al. 2013; Liao et al. 2006; Sadilek and Kautz 2010). Two human beings travelling from home to work and then to a restaurant may visit utterly different locations in real physical space. In an abstract activity space these locations nevertheless intersect. Semantically two homes, two workplaces, and two restaurants are the same: locations for living, working, and eating. Hence in abstract space, a qualitative analysis of the two paths is feasible.

**Figure 4.**
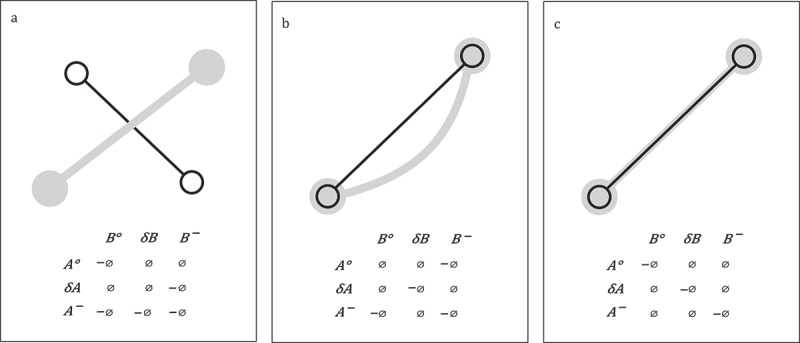
Three examples for a 9-intersection relation between two paths based on Egenhofer and Herring (1991).

Figure 4 shows three examples for 9-intersection relations between two paths. In (a) the paths entirely intersect; in (b) the start end positions intersect, whereas the interior of the paths do not; in (c) only the interior of the paths intersect. For reasons of better readability 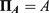 and 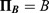. In the matrix, the empty set (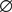) indicates that the respective elements of the paths (interior, exterior, or boundary) do not intersect; its negation (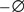) denotes an intersection. For all additional relations, see Egenhofer and Herring (1991).

Versichele et al. (2012) install a system of static Bluetooth scanners to monitor the movement of visitors of a cultural festival in the city of Ghent. Visitors who follow the same (sub-)path from one Bluetooth scanner to the other could be interpreted as one of the 9-intersection relations, i.e. relation (c) in Figure 4.

Gruteser and Hoh (2005) equip students at the State University of New Jersey with GPS receivers and monitor their movement. One of their (obvious) observations is that the students’ paths *intersect* at the University Campus (cf. relation (a) in Figure 4).

For paths that occur in a non-discretized space, quantitative comparison measures play a far more important role, especially in the field of (time-relaxed) trajectory clustering. Trajectory clustering finds those objects that move close to one another in space (time-relaxed) or space–time (time-aware). In order to quantify spatial closeness, clustering relies on a specific distance measure. We distinguish between two different types of distance measures for paths: either the similarity measures account for the entire path (global measures) or only some segments of the path (local measures).

##### Global path similarity

A simple and straightforward measure for comparing two paths is the *Euclidean distance* between the two pairs of respective boundary positions, i.e. the distance between the two origins (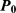) and the two destinations (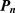). Rinzivillo, Pedreschi, et al. (2008) refer to the average of these as the *common source and destination distance. Common source and destination distance* is computationally fast. In a similar manner, the distance function *k points* calculates the average Euclidean distance between several spatial positions along the path. These spatial positions are referred to as checkpoints (Rinzivillo, Pedreschi, et al. 2008); *k points* require *k* checkpoints. Hence, the two paths are split into *k–1* segments; each segment consists of equally many spatial positions. The number of positions per segment is not necessarily the same for both paths. In general, *k points* is computationally fast; the number of checkpoints controls the computational costs. Rinzivillo, Pedreschi, et al. (2008) apply *common source and destination distance* as well as *k points* to cluster vehicle GPS data in space.

If every (recorded) spatial position of a path is considered a checkpoint, the resulting distance is referred to as the *Euclidean distance* between two paths (Zhang, Huang, and Tan 2006). Euclidean distance requires two paths to have the same number of spatial positions. Generally, it is of quadratic computational complexity. Cai and Ng (2004) propose a computationally fast approximation of *Euclidean distance* between two paths. They apply it to retrieve the similarity of hockey players’ movement on the pitch.

The *common route distance* (Andrienko, Andrienko, and Wrobel 2007) continuously searches two paths for positions that spatially match, that are within a certain distance threshold of each other. It calculates the mean Euclidean distance between matching positions and a penalty distance for positions that do not match. Hence, its computational complexity is also quadratic. *Common route distance* can handle incomplete and faulty data, due to its relative insensitivity to outliers. As it does not satisfy the symmetry axiom, *common route distance* is not a metric. However, it becomes a metric if modified to 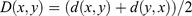. Andrienko, Andrienko, and Wrobel (2007) apply *common route distance* to a truck data set collected in the city of Athens and cluster trucks that follow similar paths.

Junejo, Javed, and Shah (2004) apply a distance function based on *Haussdorff distance* for finding similar paths of people moving in video surveillance scenes. For two spatial paths 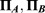 the *Haussdorff* distance checks which position of path 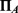 is farthest from path 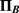 and which position of 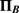 is farthest from 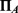 (Chew et al. 1997). These do not necessarily have to match. From the two candidate positions the one that is biggest constitutes *Hausdorff* distance. *Hausdorff* distance is a non-metric similarity function. It becomes a metric if modified to 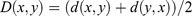.

Pelekis et al. (2012) propose the *locality in between polylines* (*LIP*) distance function. *LIP* calculates the area between two paths on a Cartesian plane; it is used by the authors for clustering vehicle GPS data in space. *LIP* may express the global similarity between two paths as well as the local similarity. LIP is comparably fast and has quasilinear computational complexity. It is not a metric but becomes one if modified to 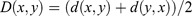


Lin and Su (2005) propose a distance measure between two paths called the *one-way distance* (*OWD*). *OWD* from the path 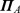 to the path 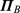 is defined as follows: first, the integral of Euclidean distances between all positions 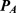 of 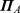 and their corresponding position in 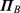 is calculated. Corresponding positions are those that are closest in space. Then, the integral is divided by the cumulative length of the path 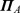. As the *OWD* distances from 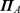 to 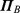 and from 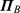 to 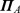 differ, *OWD* is not a metric. It becomes a metric if modified to 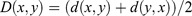. *OWD* is used by Lin and Su (2005) to perform similarity search on simulated random walk trajectory data. The computational complexity of OWD is low (i.e. quasilinear).

##### Local path similarity

For local path similarity, a path is considered a segment of simple lines, where one line connects consecutive spatial positions. Rather than the entire path, some sub-segments comprising one or several lines are analyzed for similarity, whereas others are simply not considered.

Lee, Han, and Whang (2007) combine three types of distance measures to assess the similarity of two lines: *angular distance*, *perpendicular distance*, and *parallel distance.* Let 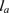 and 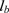 be two lines, where 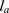 is longer than 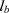. *Angular distance* is defined as 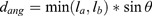, where 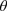 is the angle between the two lines. Consider that the start and end position of the shorter line are projected onto the longer one. Then the *perpendicular distance* is the Lehmer mean from the start and end position to their respective projection points on the longer line: 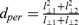 . The *parallel distance* is the minimum of the two distances from the projection point to the end point parallel to the longer line: 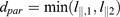. Lee, Han, and Whang (2007) utilize their approach for clustering hurricane data and radio-telemetry data of animal movement in quasilinear time. For more information on *angular distance*, *perpendicular distance*, and *parallel distance* see *Chen, Leung, and Gao (2003)*.

Bashir, Khokhar, and Schonfeld (2003) use *principal component analysis* (*PCA*) to cluster matching paths in video retrieval scenes. Their approach concatenates the spatial positions of a path into a one-dimensional signal. Then, *PCA* filters out those coefficients of the path that are most important, i.e. that contribute most to the path’s variance. In a last step, the Euclidean distance between these remaining coefficients is calculated.

#### Travelled distance and range

Travelled distance and range are derived measures of movement. Hence, the topological relations of comparison are given by the *relational* operators ‘=’ (equal travelled distance/range), ‘<’ (shorter travelled distance/range), and ‘>’ (longer travelled distance/rang*e*). A quantitative means of comparison is the *difference between travelled distance/range*.

Travelled distance and (home) range play an important role in ecology and research on human mobility. Merrick and Loughlin (1997) compare the travelled distance and the home ranges of foraging stellar sea lions in Alaska. Mate, Nieukirk, and Kraus (1997) track the movement of whales in the North Atlantic and compare their travelled distances. Tøttrup et al. (2012) record the annual migration cycle of red-backed shrike from Europe to Africa and find that during spring migration the birds travel a 1/5 *longer* distance, as they take a detour over the Persian Peninsula. González, Hidalgo, and Barabási (2008) study the spatial occurrence of mobile phone calls of mobile phone subscribers. They calculate the step size (or travelled distance) between each two calls of one user and find that overall human displacement is highly predictable. Moreover, they calculate and compare the mobile phone users’ radii of gyration, a measure that corresponds to a range from the trajectories’ center of gravity.

#### Heading

Heading indicates the relative direction toward which an object moves. If heading is interpreted as an angular measure, it may be compared in a topological manner with the three relational operators: ‘*=*’ (same angle), ‘<’ (smaller angle), and ‘>’ (bigger angle). Moreover, the *difference between angles* can be calculated. This difference can be interpreted in a qualitative manner: if object *A* moves at a *difference* of around 180° with respect to *B*, the two objects are said to move *into opposite directions*. If relative direction is interpreted in the sense of a cardinal direction (cf. Frank 1996) two relations suffice for comparison: ‘*=*’ (same cardinal direction) and ‘
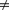
’ (different cardinal direction). Additionally, the qualitative relation in *opposite cardinal direction* may also be used.

Melnychuk, Welch, and Walters (2010) track migrating salmon and study their heading after entering the ocean. They find that salmon from two different rivers tend to migrate into *opposite cardinal directions:* the one swim North, the others South. Laube and Imfeld (2002) and Laube, Imfeld, and Weibel (2005) use heading as one parameter in their *REMO* analysis concept. They apply *REMO* to caribou GPS data in order study their behavior. They find that during spring the caribous head mostly to North and Northeastern direction, whereas in summer they rather tend toward South and East.

Pelekis et al. (2007) develop a computationally fast measure to compare the consecutive headings of two moving objects along their path. The differences between these result in the overall *directional similarity* between the two movements. They apply their algorithm to find similar vehicle GPS trajectories.

#### Shape

Shape describes how a moving object ‘winds’ its way through a spatial reference system. Shape similarity is expressed as a qualitative (topological) or quantitative relation of the shape parameter under consideration, i.e. *sinuosity*, *curvature*, *tortuosity*, *curviness*, and *fractal dimension.* Without neglecting the semantic differences between these, we henceforth use *sinuosity* as a proxy for all. Again, the relational operators ‘=’ (equal sinuosity), ‘<’ (smaller sinuosity), and ‘>’ bigger sinuosity represent the topological relations, whereas a quantitative relation is given by the *difference* between two sinuosity measures.

In biology the sinuosity of an animal’s path is a key measure for classifying searching behavior. It helps researchers to distinguish between a planned, oriented, and effective behavior (low sinuosity) and a random search behavior (high sinuosity) (Benhamou 2004). Focardi, Marcellini, and Montanaro (1996) study the movement of deer and infer different foraging behavior from the sinuosity of their paths. The degree of winding of a path is also used to reason about human behavior. Enguehard, Devillers, and Hoeber (2011) calculate the fractal dimensions of ship trajectories in the Atlantic Ocean in order to infer similar fishing activities.

In addition to the above-mentioned comparison measures, Vlachos, Gunopulos, and Das (2004) propose a quantitative distance measure to assess the similarity of spatial shapes. First, the authors map each position difference vector of a path to a rotation-invariant space, where one dimension represents the direction and the other the length of the vector. In this space, *Dynamic Time Warping* (*DTW*) (see section ‘Spatiotemporal trajectory’) is applied to find shapes of similar form. This measure is not affected by rotation, scaling, and transformation. Vlachos, Gunopulos, and Das (2004) use their approach to find similar letters in handwriting trajectories.

A slightly different approach is presented by Yanagisawa, Akahani, and Satoh (2003). They interpret the paths of two moving objects as a series of consecutive position difference vectors independent of an absolute reference point in space. Then they calculate the *squared Euclidean distance* between these, and consequently, determine the shape similarity of the two movements. Yanagisawa, Akahani, and Satoh (2003) test their measure on simulated trajectory data.

### Spatiotemporal similarity measures

#### Spatiotemporal position

The topological relations of two spatiotemporal positions can be inferred from those of time instance and spatial position. Two spatiotemporal positions either *intersect* or do *not intersect*. Calabrese et al. (2010) analyze sport events and mobility in cell phone networks in the city of Boston. During an event, such as a baseball game, many mobile phone users are found in the same mobile phone cell at the same time. Hence, their spatiotemporal positions *intersect*.

In order to compare two spatiotemporal positions quantitatively, three types of measures may be applied: purely spatial measures (e.g. *Euclidean distance*), purely temporal measures (e.g. *temporal distance*) and spatiotemporal measures (e.g. *Euclidean distance* and *temporal distance*). Spatial measures, on the one hand, compare spatiotemporal positions only with respect to space and neglect time. Hence, all quantitative measures for comparing spatial positions apply. Temporal measures, on the other hand, consider time, but neglect space. Therefore, the quantitative measures for comparing time instances apply. Spatiotemporal measures consider both, distance in time and space. Neglecting either space or time does not mean that they do not matter for analysis; rather the opposite is true. Time can only be neglected, if the two objects under comparisons exist at the same time. Consequently, space can be neglected, if the two objects attain the same spatial positions. Imagine we compare the spatiotemporal positions of stopover sites during bird migration. If two birds make a stopover at the same time, a simple *spatial distance* function suffices to assess the spatiotemporal similarity of the stopover sites. If the two stopover sites spatially *intersect*, *temporal distance* expresses the similarity between these.

For practical applications, *spatial measures* between spatiotemporal positions (and also trajectories) are most important. One example of these is the *k-nearest neighbor search*. In general, a *nearest neighbor (NN)* algorithm finds the one object in a set of query objects that is closest to a reference object. This object is denoted the *NN*. In the field of movement analysis, *NN search* is widely used to find the nearest static neighbors of a moving reference object, e.g. the nearest gas station from a car in a road network (Song and Roussopoulos 2001), or the nearest moving neighbor from a static reference object, e.g. the closest taxi unit from a costumer’s location. Frentzos et al. (2007) also propose a methodology for finding the nearest moving neighbor of a moving reference object, e.g. the nearest moving conspecific of a foraging animal.

**Figure 5.**
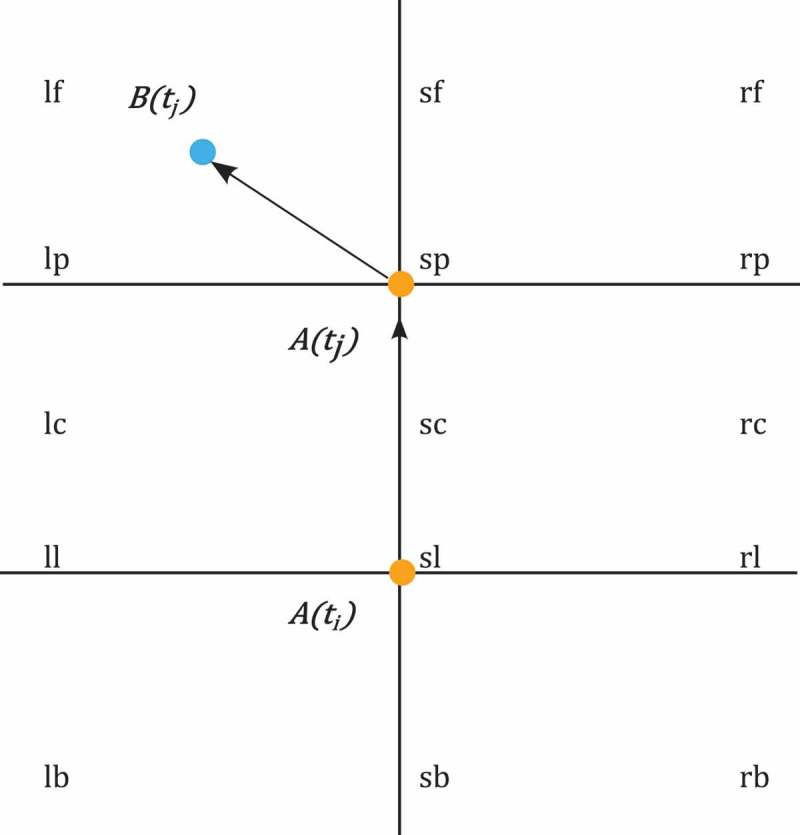
Double cross calculus (based on Freksa 1992).

In addition to *spatial distance*, we can relate two spatiotemporal positions with respect to *spatial direction*. *Double cross calculus* (Freksa 1992) is a topological measure that uses two consecutive spatiotemporal positions of a moving object *A* to partition space into 15 qualitative regions. The resulting double cross then describes the current location of a second moving object *B* relative to *A*’s position and current movement. In Figure 5, the moving object *A* (orange dot) changes its position from time 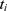 to 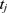. Object *B*’s relative position to that movement is *lf* (left front). Schiffer, Ferrein, and Lakemeyer (2006) use a qualitative partitioning of space similar to that of the *double cross calculus* to plan the movement of agents of a robot football squad and find successful strategies for scoring goals. The computational complexity of *double cross calculus* is discussed in Scivos and Nebel (2001).

The *qualitative trajectory calculus* (*QTC*) (Van de Weghe 2004) is similar to the *double cross calculus*. It compares the current movement of object *A* with respect to that of object *B*. In its basic form *QTC* has four qualitative primitives (Van de Weghe, Kuijpers, et al. 2005b). The first primitive describes whether *A* moves *away from B*, *toward B*, or *remains stable with respect to B*; the second primitive whether *B* moves *away from A*, *toward A*, or *remains stable with respect to A*. The third and fourth primitives describe in which relative direction (left, right, stable) the two objects move with respect to one other. Hence, *QTC* converts relative direction and distance information between two objects at one specific spatiotemporal position into a qualitative measure. In contrary to traditional approaches of qualitative spatial reasoning *QTC* allows for formalizing dynamic changes between two objects. Van de Weghe, Cohn, et al. (2005) apply *QTC* to describe overtaking events between two cars, i.e. object *A* starts behind object *B*, pulls out, overtakes *B* and finish in front of it.

#### Spatiotemporal trajectory

To the best of our knowledge, in literature, there are no genuine methods that compare entire trajectories in a topological manner. However, there are some approaches that are applicable to (sub-)trajectories with certain constraints. In an extension of the 9-intersection model *Kurata and Egenhofer (2006)* model the relations of directed lines. Directed lines are non-intersecting line segments in two-dimensional space. They comprise a head (i.e. the end point), a tail (i.e. the star point), and a body (the interior). Thus, trajectory segments that do not intersect may be interpreted as directed lines. *Kurata and Egenhofer (2006)* define 68 *head–body–tail relations between two directed lines*. These are capable of modeling abstract movement patterns such as two moving objects *splitting* and *meeting.* In another work Kurata and Egenhofer (2007) extend this model to relations between directed lines and regions. Amongst other things these allow for describing a moving object entering, passing through or leaving a certain geographical area.

Besides *head–body–tail relations*, *QTC* (cf. section ‘Spatiotemporal trajectory’) allows for qualitative reasoning at single spatiotemporal positions *along* the trajectory. Other topological approaches (i.e. Gerevini and Nebel 2002; Wolter and Zakharyaschev 2000) are not sufficiently capable of handling trajectories.

In contrast to this, quantitative trajectory similarity measures are abundantly used in literature. Quantitative trajectory similarity is closely related to the problem of time-aware clustering. Time-aware clustering finds those objects that move close to one another in space and time. In literature, various terms have been coined for time-aware clustering: some authors refer to it as trajectory clustering (Buchin et al. 2008; Nanni and Pedreschi 2006), as clustering moving objects (Li, Han, and Yang 2004), identifying flocks (Benkert et al. 2008; Wachowicz et al. 2011), convoys (Jeung et al. 2008), moving clusters (Kalnis, Mamoulis, and Bakiras 2005), or swarms (Li et al. 2010). Though the different connotations of all of these terms are generally acknowledged for – i.e. some analyze entire trajectories, whereas others concentrate on sub-trajectory similarity – they are nevertheless often used interchangeably and ground on one common denominator: objects moving close in space and time.

In movement analysis, trajectories are often interpreted as a series of positions ordered in time. Hence, methods for assessing the similarity of time series are applied also for trajectories. According to Ding et al. (2008) and Saeed and Mark (2006), similarity measures for time series can be grouped into three types: lock-step measures, elastic measures, and developed based measures. Similar to path similarity, trajectory similarity measures can also apply for the entire trajectory (global measures) or sub-trajectories (local measures). These are, however, not used as the main criteria for the following classification, but mentioned where necessary.

##### Lock-step measures

**Figure 6.**
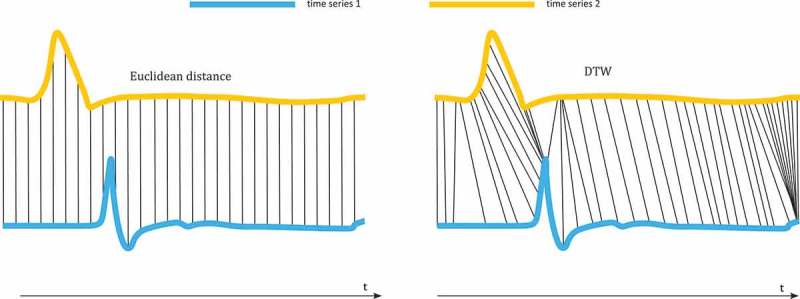
Lock-step measure (Euclidean distance) and elastic measure (DTW).

*Lock-step measures* compare the *i*th element of one time series *A* to the *i*th element of another time series *B* (see also Figure 6). The most straightforward distance measure to compare two elements is *Euclidean distance*. Lock-step distance measures are sensitive to noise and misalignments in time, since the mapping between the elements of two time series is fixed. Nanni and Pedreschi (2006) propose a lock-step distance measure for clustering trajectories. They calculate the sum of all distances between two spatiotemporal positions of two objects matching in time. Then they divide this distance by the duration that the two objects move together. A similar approach for assessing the dissimilarity of two trajectories (*DISSIM*) is presented by Frentzos, Gratsias, and Theodoridis (2007). Here, the sum of all *Euclidean distances* equals the dissimilarity of the trajectories. In addition to that, a local trajectory similarity measure based on Euclidean distance is presented by Buchin et al. (2009).

##### Elastic measures

*Elastic measures* either do not consider all elements in the time series for comparison, or they allow a comparison between elements that do not match in time (see also Figure 6).

*Dynamic time-warping* (*DTW*) is a similarity measure between two sequences which may vary in time or speed. The sequences are ‘stretched’ or ‘compressed’ non-linearly in the time dimension to provide a better match with another time series (Berndt and Clifford 1994; Keogh and Pazzani 2000). The technique has originated in speech recognition. Here, phonemes of an input expression may differ in length and speed from the phonemes in a reference expression. *DTW* allows for aligning the input and reference expression in an optimal way. DTW is particularly suited to matching sequences with missing information. Little and Gu (2001) apply *DTW* to trajectories from video sequences. Fu et al. (2008) combine *DTW* and *uniform scaling* to a *Scaled Warped Matching* technique (*SWM*). Uniform scaling stretches a time series in a uniform manner. Amongst others the researchers use *SWM* to assess the similarities of trajectories of high jumpers. In general, *DTW* is performed in quadratic time.

The *LCSS* (Vlachos, Kollios, and Gunopulos 2002) finds the longest subsequence (cf. Bollobás et al. 1997) that is common in two trajectories 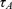 and 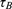. A subsequence is an alignment of elements that occurs in both sequences given that the order of the remaining elements is preserved. In the case of applying *LCSS* to trajectories, temporally matching spatial positions are used as elements; the spatial proximity between these determines whether or not two elements are equal. Trajectories share a common element if the Euclidean distance between two of their spatial positions is less than or equal to a threshold. *LCSS* is performed in quadratic time. Vlachos, Kollios, and Gunopulos (2002) apply *LCSS* to cluster animal GPS data.

*Time steps* is a distance measure for trajectories similar to *k-points* for paths (described in section ‘Spatial path and line’). In contrast to *k-points* a specific temporal distance lies between each two checkpoints. *Time steps* is computationally fast; the temporal distance defines the computational costs. Rinzivillo, Pedreschi, et al. (2008) apply *time steps* to cluster vehicle GPS data.

The *common route and dynamics distance* stems from the *common route distance* described in section ‘Spatial path and line’. The function regards two positions to match if they are spatially close and attained at similar relative times. Relative time starts at the time instance that marks the beginning of each trajectory. Hence, *common route and dynamics* analyzes whether the trajectories are spatially similar and travelled in a similar dynamic progression. Andrienko, Andrienko, and Wrobel (2007) use *common route and dynamics* to cluster vehicle GPS data.

Another similarity measure between two trajectories is the Fréchet distance. An intuitive definition of the Fréchet distance is presented by Aronov et al. (2006). A person and his dog move next to each other, the person keeps the dog on the leash. Both person and dog are free to choose their spatial path and their leash. The Fréchet distance denotes the minimum length of the leash that ensures that the person and the dog are always connected. Fréchet distance is computationally expensive. It is applied by Buchin, Buchin, and Gudmundsson (2010) to globally cluster bicycle GPS data and simulated random walk data and by Buchin et al. (2008) to locally cluster pedestrian GPS data.

##### Developed based similarity measures

*Developed based* similarity follows the *edit distance* concept, initially proposed by Levenshtein (1966). Edit distance counts the number of operations required to transform one string *A* into another string *B*. The lower this number is, the more similar the strings are. Chen, Tamer Özsu, and Oria (2005) propose a distance measure for trajectory similarity search based on *edit distance* called *edit distance on real sequences* (*EDR*). Given two trajectories 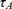 and 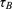, *EDR* (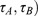 represents the number of insert, delete or replace operations, that change the spatiotemporal positions in 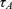 such that that they match those in 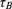. Two spatiotemporal positions are said to match if their coordinate tuples are similar according to a certain threshold. *EDR* is a non-metric similarity function. It is shown to be very robust with respect to outliers; its complexity for comparing two trajectories is quadratic. Chen, Tamer Özsu, and Oria (2005) test *EDR* on simulated random walk data.

Other examples for developed based distances are *Hamming distance* (Hamming 1950) or *Jaro–Winkler* distance (Winkler 1999). These have, however, not been applied to trajectory data.

In addition to the above-mentioned methods, others have been developed to compare time series (Keogh and Kasetty 2003). These methods include the Sequence Weighted Alignment model (Swale) (Morse and Patel 2007), Spatial Assembling Distance (SpADe) (Chen et al. 2007), and similarity search based on Threshold Queries (TQuEST) (Aßfalg et al. 2006). It has, however, not been tested if these methods are applicable to trajectories.

##### Other distances measures

In addition to measures that explicitly assume trajectories as time series, there are such that ground on other concepts. These are listed here.

*Lifeline distance* (Sinha and Mark 2005) assumes that objects remain static for a sufficiently long time and then abruptly change their location, such as a person moving from one mobile phone cell to another. *Lifeline distance* represents the temporally weighted average of successive distances between the two entities. Hence, *lifeline distance* is not an appropriate similarity measure for moving objects that continuously change their position. Moreover, it is not a metric.

Porikli and Haga (2004) propose a distance function between two trajectories based on the *Hidden Markov model* (*HMM*). The positions along a trajectory are used as observations from which the *HMM* is inferred. The *HMM* is the previously hidden sequence of states of the object. Then the likelihood of the trajectory to its own *HMM* is compared to the likelihood to fit the *HMM* of another trajectory. This difference constitutes the *HMM distance* between the two trajectories. The authors use HMM to find outliers in video data of vehicle trajectories. Apart from spatiotemporal positions, *HMM distance* may also fall back on speed, acceleration and other qualitative observations of movement (color, size of the object) to infer current and future *HMM* states. *HMM distance* is computationally expensive, i.e. it is performed in polynomial time.

Pelekis et al. (2007) extend *the LIP* distance for comparing spatial paths to a *spatiotemporal LIP* distance (*STLIP*). *STLIP* allows for comparing two trajectories in quasilinear time. The authors apply their measure to cluster GPS vehicle data.

#### Velocity and acceleration

For comparing the qualitative (topological) relations of speed and acceleration (scalar) the following relational operators are used: ‘*=*’ (same speed/acceleration), ‘<’ (slower/lower acceleration), and ‘>’ (faster/higher acceleration). An extension of QTC (see section ‘Spatiotemporal position’) incorporates these (Van de Weghe 2004); it allows for defining whether object *A* moves *faster, slower, or at the same speed* compared to object B and whether object A *accelerates faster, slower, or equally*. The *difference in speed*/*acceleration* is the respective quantitative measure.

Pelekis et al. (2007) develop a *speed-pattern based similarity* measure. They interpret two movements as speed curves over time (or space) and calculate the similarity between these as an average of all respective *differences in speed* in quasilinear time. The authors apply their approach to cluster GPS trajectories of vehicles.

In general, the comparison of the dynamics of movement plays a crucial role for mode detection (Zheng, Li, et al. 2008, Zheng, Liu, et al. 2008). Zheng et al. (2010) compare speed and acceleration along multimodal GPS tracks to typical walking speed and acceleration. Thus, they partition the track into (probable) walking segments and non-walking segments. From these, further modes of transport are inferred.

Dodge, Laube, and Weibel (2012) present a different approach for comparing the dynamic similarity of movement. Their *normalized weighted edit distance* (*NWED*) translates speed and acceleration information along the movement into a sequence of different symbolic representation. One such representation may, for example, correspond to a slow speed without acceleration, another one to high and fluctuating speed. Then, *edit distance* is applied to these symbolic sequences, in order to find movement that has a similar dynamic behavior. Dodge, Laube, and Weibel (2012) utilize *NWED* to find similar instances in hurricane data and vehicle GPS data. *NWED* allows comparing two movement trajectories in quadratic time.

The above-mentioned relational operators also apply for comparing *change of heading*, if it is transferred into an angular measure. Then this parameter is mostly termed *turning angle*; its relations are given by ‘=’ (same turning angle), ‘*<*’ (smaller turning angle), ‘>’ (bigger turning angle). The *difference in turning angle* represents the respective quantitative measure.

Turning angle is an important parameter in the analysis of animal movement. It is closely related to shape measures (such as the *sinuosity* of a path) and therefore also allows for inferring the behavior of the animal under study. Waddington (1979) analyzes the turning angle of a bee’s flight to reason about different foraging behavior. Müller and Wehner (1988) study the foraging movement of ants. They find that ants move from the anthill to their foraging grounds with *bigger turning angles*, compared to their homebound trajectories, and follow that their homebound movement is much more determined.

## Summary and conclusion

In this paper we collect measures that asses the similarity of movement. We first decompose movement into its physical quantities in time, space, and space–time. For each of these, we review primary and derived similarity measures. We show the main purpose of each measure and its computational complexity and find empirical research in the field of geographic information science and beyond where the measure is applied. Table 1 synthesizes the results and shows the reviewed similarity measures, their characteristics, and movement parameters they relate to.

**Table 1.** Movement similarity measures and their characteristics.

Similarity measure	Movement parameter	Purpose	Primary/ Derived	Topological/ Quantitativ	Complexity
Allen’s temporal logic	Time instance, time interval	des, beh	P	T	–
Temporal distance	Time instance, time interval, spatiotemporal position	des, beh	P	Q	L
Relational operators	Duration, distance, range, heading, shape, speed, acceleration, change of direction	des, beh	D	T	L
Quantitative difference	Duration, distance, range, heading, shape, speed, acceleration, change of direction	des, beh	D	Q	L
9-intersection	Spatial position, path	des, beh	P	T	–
Euclidean distance	Spatial position, path, spatiotemporal position, trajectory	clust, sim,	P	Q	M
Minkowski distance (e.g. Manhattan distance)	Spatial and spatiotemporal position	des	P	Q	L
Distance along curved surface	Spatial and spatiotemporal position	des	P	Q	L
Network distance	Spatial and spatiotemporal position	des	P	Q	M
Relative direction	Spatial and spatiotemporal position	des	P	–	L
Cardinal directions	Spatial and spatiotemporal position	des	P	Q	L
REMO	Heading	beh	D	Q	–
Common source and destination	Path	clust	P	Q	L
Common route	Path	clust, beh	P	Q	M
Haussdorff	Path	clust, out	P	Q	H
k points	Path	clust	P	Q	L–M
OWD	Path	sim	P	Q	L
LIP	Path	clust	P	Q	L
PCA	Path	clust	P	Q	L
Combined angular distance perpendicular distance and parallel distance	Line	sim	P	Q	L
Directional similarity	Heading	sim	D	Q	L
Head–body–tail relations	Line, (sub-)trajectory	des	P	T	–
DTW	Trajectory, shape	clust	P, D	Q	M
Squared Euclidean	Shape	sim	D	Q	M
Double cross calculus	Spatiotemporal position	des	P	T	–
QTC	Spatiotemporal position, speed, acceleration	des, beh	P, D	T	–
k-nearest neighbor	Spatiotemporal position	sim	P	Q	–
LCSS	Path, trajectory	clust, sim	P	Q	M
Time steps	Trajectory	clust	P	Q	L–M
Common route and dynamics	Trajectory	clust, beh	P	Q	H
Fréchet	Trajectory	clust	P	Q	H
EDR	Path, trajectory	sim, clust	P	Q	M
Lifeline distance	Trajectory	clust	P	Q	–
HMM	Spatiotemporal position, trajectory	out	P	Q	H
STLIP	Trajectory	clust	P	Q	L
Speed-pattern based similarity	Speed	clust	D	Q	L
NWED	Speed, acceleration	sim, clust	D	Q	M

Note: Purpose: sim *= similarity search*, clust = *clustering*, beh = *behavior analysis*, des *= description*, out = *outlier detection*; Primary/Derived: *P* = *primary*, *D* = *derived;* Topological/Quantitative: *T* = *topological*, *Q* = *quantitative;* Complexity: *L* = *low*, *M* = *medium*, *H* = *high.*

In the review we identify a lack of topological measures for comparing (entire) spatiotemporal trajectories. To the best of our knowledge these have not been proposed or discussed in literature. Possible reasons for this are further discussed in section ‘Discussion and future work’. The opposite holds true for quantitative trajectory similarity. These are exhaustively discussed in literature.

## Discussion and future work

In this paper we structure movement similarity measures according to the movement parameter they compare. Some similarity measures may, however, not be fully assigned to a single parameter. An example for such is the *dynamics aware similarity method of trajectories (Trajcevski et al. 2007)*. This measure assesses the shape similarity of two trajectories, together with speed similarity. Hence, it would most suitably qualify as a measure for comparing *spatiotemporal shape*, which we do not define as a movement parameter.

Other similarity measures are capable of comparing more than one parameter. Especially, quantitative methods for spatial paths and spatiotemporal trajectories are often used interchangeably. To name two examples, *LCSS* and *EDR* can compare spatial paths and spatiotemporal trajectories. We believe that this stems from the interchangeable use of the expressions path and trajectory, on the one hand, and the fact that time is most naturally used to order respective positions along a path, on the other hand. For example, *time step*s analyzes path similarity irrespective of time, but requires time stamps to define which elements of the paths are to be compared.

Another clearly hybrid similarity measure is *common route and dynamics distance*. It does not require two objects to have similar trajectories, but they need to travel their paths in a similar temporal progression, successive spatial positions have to be reached at similar relative times.

In spite of these shortcomings, we still believe that our classification allows for a structured overview on different aspects of movement similarity, and a better understanding on how movement similarity is interpreted and implemented in geographic movement analysis.

A topic that has only been discussed briefly in this paper is that of the possible application fields for topological similarity of movement and the question: when does topological comparison of movement actually *make sense*? Time intervals, paths, and trajectories can only share the same *positions* in a reference system, if this reference system realistically allows for that, i.e. by consisting of discrete time bins, spatial cells, or a spatiotemporal derivative of both. Hence, it seems quite logical that our review reveals a lack of measures for assessing the topological relations of two spatiotemporal trajectories. We argue that such a measure may still be relevant. Furthermore, we believe that it may be derived in a straightforward manner from Egenhofer’s 9-intersection model for paths together with a simple temporal extension. The temporal extension specifies which ‘position in time’ the respective intersecting elements of the matrix have.

**Figure 7.**
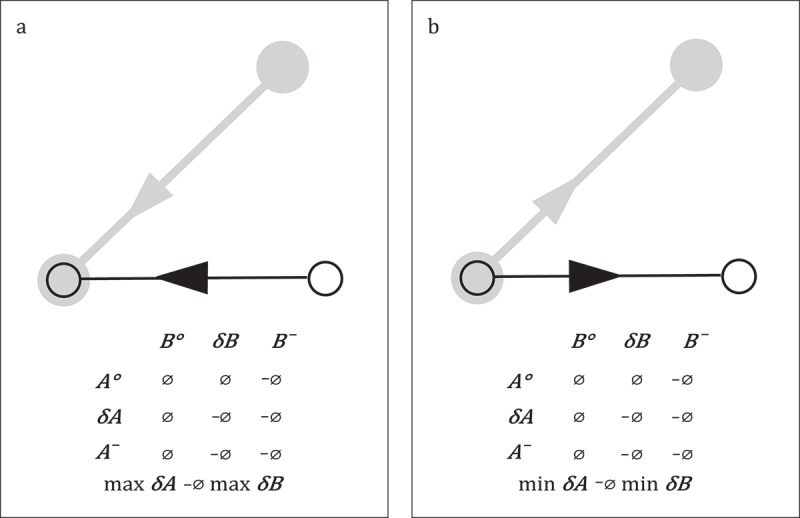
Topological relation for two converting and dispersing trajectories.

Figure 7 shows one example for a possible qualitative trajectory measure. In (a) the temporal extension 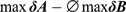 denotes that the end points of the trajectory intersect; in (b) 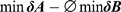 denotes that the start points intersect. Together with the 9-intersection relation, these describe in a formal way that two trajectories (a) convert or (b) disperse. Conversion relates to a movement to a common destination, dispersion to a movement away from a common origin (Dodge, Weibel, and Lautenschütz 2008). In future work we want to further elaborate on these ideas.

Last but not least we observe that certain primary similarity measures result in a similarity of derived measures. If two time intervals intersect, they have the same duration; similarity of spatial path results in a similar travelled distance and shape; trajectory similarity means similar speed and acceleration, to name but a few obvious similarity dependencies. A systematic analysis of all dependencies between different similarity measures is out of the scope of this paper, but is an interesting topic for future work.

## Note

1. 
http://www.gps.gov/systems/gps/performance/accuracy/
